# Establishing a risk assessment framework for point-of-care ultrasound

**DOI:** 10.1007/s00431-021-04324-4

**Published:** 2021-11-30

**Authors:** Thomas W. Conlon, Nadya Yousef, Juan Mayordomo-Colunga, Cecile Tissot, Maria V. Fraga, Shazia Bhombal, Pradeep Suryawanshi, Alberto Medina Villanueva, Bijan Siassi, Yogen Singh

**Affiliations:** 1 Department of Anesthesiology and Critical Care Medicine, Children’s Hospital of Philadelphia and Perelman School of Medicine, Philadelphia, PA USA; 2 Division of Pediatrics and Neonatal Critical Care, Antoine Béclère Medical Center, APHP, South Paris University Hospitals, Paris, France; 3 Pediatric Intensive Care Unit, Department of Pediatrics, Hospital Universitario Central de Asturias, University of Oviedo, Oviedo, Spain; 4 Department of Pediatrics, Chêne-Bougeries, Clinique des Grangettes, Geneva, Switzerland; 5 Department of Pediatrics, Children’s Hospital of Philadelphia and Perelman School of Medicine, Philadelphia, PA USA; 6 Division of Neonatal and Developmental Medicine, Stanford University School of Medicine and Lucile Packard Children Hospital Stanford, Stanford, CA USA; 7 Department of Neonatology, Bharati Vidyapeeth University Medical College, Pune, Maharashtra India; 8 Pediatric Intensive Care Unit, Department of Pediatrics, Hospital Universitario Central de Asturias, University of Oviedo, Oviedo, Spain; 9 Department of Pediatric Cardiology, University of Southern California, Los Angeles, CA USA; 10 Department of Paediatrics, Division of Neonatology, Loma Linda University School of Medicine, CA, USA, and Depart of Pediatrics - Neonatology and Paediatric Cardiology, Cambridge University Hospitals NHS Foundation Trust, Cambridge, UK

**Keywords:** Point-of-care ultrasound (POCUS), Risk assessment, Framework, Neonates, Children

## Abstract

Point-of-care ultrasound (POCUS) refers to the use of portable ultrasound (US) applications at the bedside, performed directly by the treating physician, for either diagnostic or procedure guidance purposes. It is being rapidly adopted by traditionally non-imaging medical specialties across the globe. Recent international evidence-based guidelines on POCUS for critically ill neonates and children were issued by the POCUS Working Group of the European Society of Pediatric and Neonatal Intensive Care (ESPNIC). Currently there are no standardized national or international guidelines for its implementation into clinical practice or even the training curriculum to monitor quality assurance. Further, there are no definitions or methods of POCUS competency measurement across its varied clinical applications.

*Conclusion*: The Hippocratic Oath suggests medical providers do no harm to their patients. In our continued quest to uphold this value, providers seeking solutions to clinical problems must often weigh the benefit of an intervention with the risk of harm to the patient. Technologies to guide diagnosis and medical management present unique considerations when assessing possible risk to the patient. Frequently risk extends beyond the patient and impacts providers and the institutions in which they practice. POCUS is an emerging technology increasingly incorporated in the care of children across varied clinical specialties. Concerns have been raised by clinical colleagues and regulatory agencies regarding appropriate POCUS use and oversight. We present a framework for assessing the risk of POCUS use in pediatrics and suggest methods of mitigating risk to optimize safety and outcomes for patients, providers, and institutions.

**Table:** 

**What is Known:**
*• The use POCUS by traditionally non-imaging pediatric specialty physicians for both diagnostic and procedural guidance is rapidly increasing.*
*• Although there are international guidelines for its indications, currently there is no standardized guidance on its implementation in clinical practice.*
**What is New:**
*• Although standards for pediatric specialty-specific POCUS curriculum and training to competency have not been defined, POCUS is likely to be most successfully incorporated in clinical care when programmatic infrastructural elements are present.*
*• Risk assessment is a forward-thinking process and requires an imprecise calculus that integrates considerations of the technology, the provider, and the context in which medical care is delivered. Medicolegal considerations vary across countries and frequently change, requiring providers and institutions to understand local regulatory requirements and legal frameworks to mitigate the potential risks of POCUS.*

## Introduction

The 2020 publication of international point-of-care (POCUS) guidelines for neonatal and critical care providers identified numerous clinical POCUS applications supported by both graded level of evidence and expert opinion [1]. These guidelines build upon increasing data suggesting that, across pediatric disciplines, ultrasound imaging improves patient safety and provider procedural performance [2–9], introduces new clinical data [10, 11], expedites and changes management [12–15], and may improve outcomes [16–18]. Alongside robust adult data supporting POCUS use by clinicians in non-traditional imaging specialties, the question of *why* do we use POCUS continues to be asked and answered across clinical applications.

Although POCUS has experienced significant growth in clinical use by traditionally non-imaging based specialties, little time has been spent asking (or answering) the question “how” do we integrate POCUS in our practice? *Quality* healthcare ensures the delivery of safe and effective care, the *value* of which is assessed in the context of *cost*. *Risk* potentially impacts both the delivery of quality care and cost to providers, patients, and institutions. Frameworks for risk assessment exist across varied professional endeavors. A leading group within the National Health Service (NHS) in England recently published a risk assessment framework (RAF) to standardize methods of prospectively evaluating risks associated with clinical practice [19]. Importantly, the authors suggest that the RAF “should be tailored to the specific needs of the assessment” and is “not intended to be exhaustive.” Thus, the RAF provides a flexible and wholistic approach to *identify*, *analyze*, *evaluate*, and *manage* risk in the clinical setting (Table 1). This manuscript seeks to integrate concerns, experiences, and opinions from the literature as well as from our diverse international co-authorship within the RAF framework to assess pediatric POCUS risk.

**Table 1:** Risk assessment framework for point-of-care ultrasound

**Risk assessment step**	**Considerations**
Identify	• Describe the system including elements and interactions • Define undesired outcomes including patient, provider and institution • Identify potential contributory factors to undesired outcomes • Describe potential consequences
Analyze	• What controls are in place to identify and prevent undesired outcomes • Assess the severity and likelihood of undesired outcomes • Identify risk level
Evaluate	• Describe the risk tolerability • Identify ineffective or non-existent controls to mitigate risk • Define required actions and plan on methods of communicating results
Manage	• Develop a multidisciplinary group of experts to address risk and manage activities • Review data and develop analytic techniques for prospective risk assessment

Kaya GK, Ward JR, Clarkson PJ. A framework to support risk assessment in hospitals. Int J Qual Health Care. 2019;31:393–401

## POCUS risk: current concerns

In 2020, the European Society of Paediatric Radiology (ESPR) published a position paper on the use of POCUS by non-radiology performers. In this position paper, multiple concerns were raised regarding translation of this technology to new practice environments. Current training platforms were described as a “gimmick,” and the practice itself is suggested to result in missed diagnosis, delayed therapeutics and increased costs for families and institutions [20]. Similarly, in 2020 the Emergency Care Research Institute (ECRI), a nonprofit organization designated as an Evidence-based Practice Center in the USA providing guidance for US healthcare regulatory agencies such the Agency for Healthcare Research and Quality (AHRQ) and the Joint Commission, identified POCUS as number 2 among the top 10 greatest technology hazards within healthcare. The ECRI stated that “safeguards for ensuring that POCUS users have the requisite training, experience, and skill have not kept pace with the speed of adoption. The lack of sufficient oversight increases the potential that patients will be adversely affected by problems associated with use, or lack of use, of the technology.”[21] Thus safety commissions, regulatory agencies, as well as our very own colleagues suggest that POCUS places providers, patients, and institutions at varied risks of adverse outcomes.

The POCUS community response to these statements has been swift and pointed. Representatives from the European Society of Emergency Pediatrics (ESEP), the Ultrasound Section of the European Society for Emergency Medicine (EUSEM), and the Pediatric Emergency Medicine Point-of Care Ultrasound (P2) “respectfully disagree with the conclusions [of the ESPR statement], especially the need for further oversight from our radiology colleagues.”[22] While the ESPR position statement recommends a well thought-out curriculum for each clinical specialty wishing to perform POCUS and lists European credentialing/certification methods for undergraduates, general radiology training, and radiology subspecialization, it should be noted that these are not requirements for licensing and performing pediatric ultrasound as a radiologist in Europe [20]. The “need for credentialing non-radiologists who want to become involved in non-radiologist point-of-care [ultrasound]” should be balanced by what is expected of radiologists themselves [23]. Similarly, a perspective published by adult and pediatric POCUS experts suggest that “if these statements [by the ECRI] are used to guide the governance of POCUS use in pediatric intensive care units (PICUs), the resulting policies may be overly restrictive of a practice that actually has several potential benefits” and that, within the ECRI report, “no objective data were presented” as a basis for concerns [24].

Three publications review POCUS litigation, none of which found medicolegal cases from the *use* of POCUS [25–27]. In fact, the only cases related to POCUS arose from its *lack of use* when the technology was available. Assessing medicolegal risk, though, is a *forward-thinking* process to *prevent* harm, whether to patient, provider, or institution. Therefore, components within a conceptual POCUS RAF may not be evidence-based and specific contributors to risk (and the weight of their contribution) will be heavily influenced by the local practice setting.

## POCUS risk: identify

The *identify* phase defines the process being evaluated and the system in which the process is being performed. In this phase we must assess not only the safety of ultrasound technology itself but also how it is used by varied clinicians. In research terms, this might be referred to as assessing the *efficacy* of the technology and the *effectiveness* of its use when employed by providers. The Curie brothers discovered piezoelectricity over 140 years ago, and for over 80 years, the medical field has utilized ultrasound in clinical practice [28]. Ultrasound is also conceptually familiar to people outside of the medical profession, whether through knowledge of music, understanding methods of echolocation used by animals, or exposure to fetal assessment in pregnancy. Among technologies physicians incorporate at the bedside, there is likely a comfort with ultrasound that is shared between providers and patients.

Ultrasound is frequently cited as possessing a desirable safety profile since it does not utilize ionizing radiation for image acquisition. Risk assessment requires us to assess aspects of the technology that may result in harm. There are, indeed, safety concerns associated with the use of ultrasound including thermal bioeffects and inertial and non-inertial cavitation [29–32]. Neonates and pediatric patients, especially extremely premature infants, theoretically may be at greater risk of this non-thermal injury phenomena compared to adults, but no studies have been published demonstrating actual risk in human beings. The physical machine itself may also expose patients to unnecessary harm if not maintained appropriately. Ultrasound probes may be exposed to contaminated body fluids and present a risk of cross-transmission of pathogenic organisms [33, 34]. Finally, like any device, age and maintenance may impact machine capabilities. Aging transducer crystals and reduced processing speeds may result in diminished quality images [35]. Other electrical devices within clinical care may create artifacts within images on an ultrasound machine [36]. Thus, even the influence of the environment on the machine could result in suboptimal images for procedures and diagnostics.

The greatest risk regarding ultrasound use is from the users themselves. While literature does not identify prior litigation resulting from POCUS studies, we must acknowledge the potential for error and harm. From a procedural standpoint, ultrasound use has robust data supporting improved pediatric provider performance and patient safety and for almost 20 years has been promoted as standard of care for central vascular access by the National Institute for Health and Care Excellence [37]. For pediatric specialists performing invasive procedures, the greatest risk is likely *not* learning and employing ultrasound during these procedures.

Diagnostic POCUS carries the risk of misdiagnosis or missed diagnosis within the scope of clinical care. However, such a risk cannot be viewed solely specific to ultrasound. In fact, almost no clinical exam findings, serologic studies, or imaging modalities have 100% etiologic and/or pathophysiologic sensitivity and specificity. Our physical exam is fraught with inaccuracies, inconsistencies, and misleading data driving therapeutics and outcomes. The stethoscope, having over 200 years of integration within the practice of medicine, cannot always be trusted regardless of experience [38]. How we define risk requires contextualization within current practice and an appreciation for its benefit within pediatric and neonatal clinical care.

## POCUS risk: analyze

**Fig. 1:**
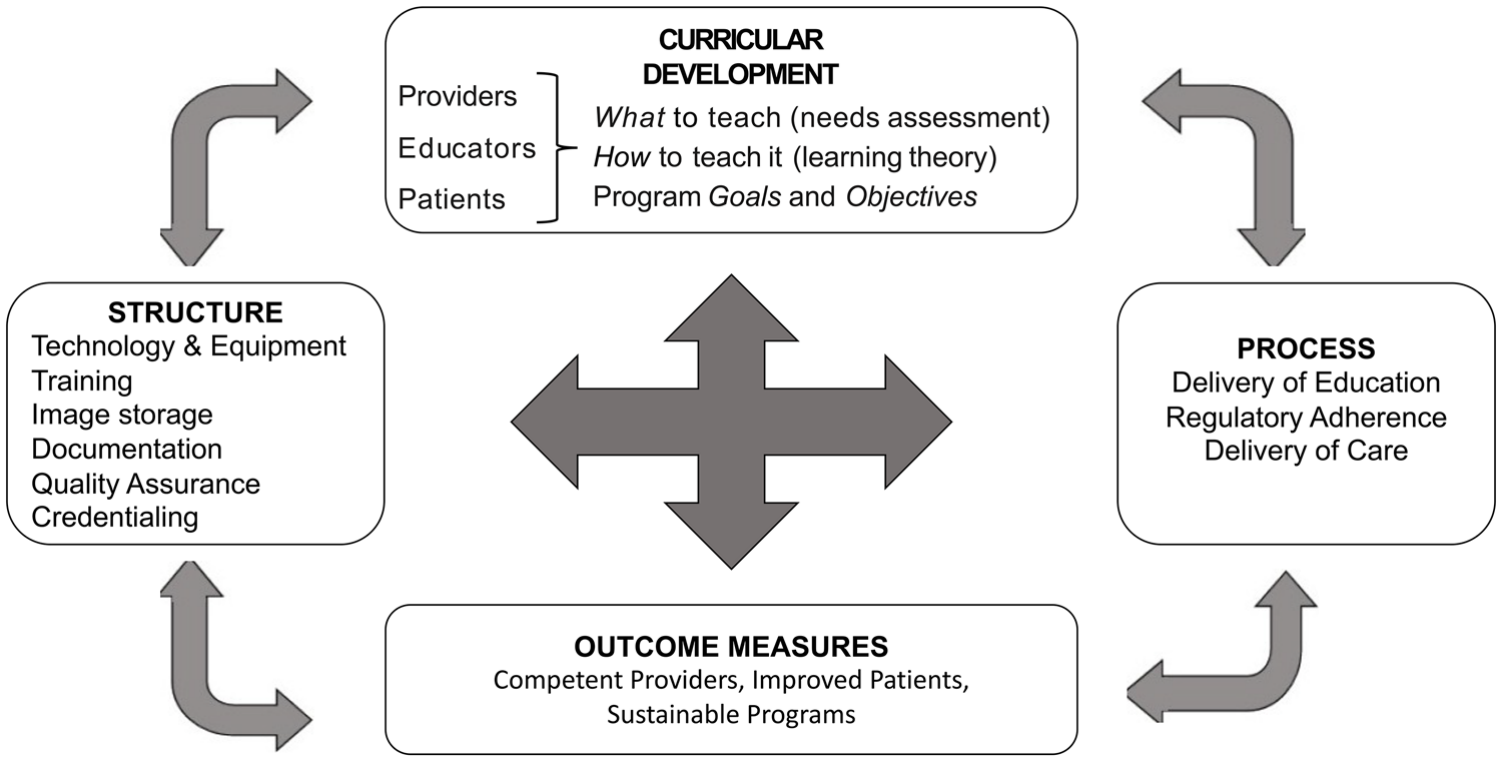
Suggested elements for program development. Infrastructural elements allow for support of effective curricular development and, through implementation processes, translates to quality care. Structure and process outcomes can be measured to assure benefit to patient, providers and institutions

This leads us to the *analyze* phase of risk assessment. What is the severity and likelihood of risk that we assume in performing ultrasound? And are there controls or methods by which we may mitigate this risk? The severity of risk is dependent upon the POCUS application performed. It is unlikely that severe harm will occur in a failed ultrasound-guided peripheral intravenous catheter insertion attempt. But imagine a POCUS evaluation of cardiac contractility interpreted as normal qualitative systolic function in a child with a clinical respiratory viral illness. What if this child is later identified to have severe systolic dysfunction on a complete echocardiogram and is subsequently diagnosed with myocarditis? Maybe the provider was correct in the initial interpretation and the cardiac dysfunction evolved over time. Or maybe the provider mis-interpreted the images and the dysfunction was present at the time of POCUS assessment. The risk to the patient is obvious, as therapies may not align with underlying pathophysiology, thereby increasing the risk of morbidity. But the risk to the provider, the department, and the institution is potentiated by factors other than the POCUS study itself. Risk increases if the provider did not receive adequate training or adequate supervision. Risk increases if the provider did not document findings. Risk increases if the provider documented an interpretation, but images were not saved and incorporated into the medical record. Risk increases if there is not a method of longitudinal assessment of physician skills. Risk increases if there are no national or institutional standards, for example, a credentialing process, for POCUS clinical integration. Thus, controls for risk involve developing infrastructural programmatic elements to standardize practice and ensure the adequate translation of training to practice (Fig. 1). Such controls are variably developed in current POCUS practice settings.

Quantifying the level of risk for a patient, a provider and an institution is an imprecise form of calculus that integrates the potential likelihood and severity of risks and associated consequences and must be balanced with potential benefit. Robust data exists in pediatrics supporting effectiveness of both procedural and diagnostic ultrasound in the hands of skilled providers, and this literature is only going to increase with time and experience [39, 40]. Strategies for mitigating risk should be viewed as opportunities to promote patient safety and optimize clinical outcomes while also serving to protect providers and their local environment. Hence, developing a robust clinical governance around use of ultrasound in clinical practice can help in minimizing such risks.

## POCUS risk: evaluate

The *evaluate* phase of risk assessment inventories current processes within the scope of mitigation strategies and solutions. This phase also evaluates the environment of practice for greater precision in assessing the degree of risk. The presence and absence of strategies and solutions are tied to local, national, and international standards. For example, training in specialty-specific POCUS is not standardized in any pediatric disciplines and there is no definition or methods of assessing for competency. Courses sponsored by societies and institutions exist to expose learners to limited applications, and there exist few practice guidelines or consensus statements regarding the role of POCUS in pediatric clinical care [1, 41–43]. Although POCUS and Targeted Neonatal Echocardiography (TNE) are certified in Switzerland by the Swiss Federation of Physicians, this is one of the very few certification processes that exist within pediatrics. Without external certification processes, nations and their individual pediatric institutions must define for themselves what entails appropriate training.

Infrastructural elements including image storage, documentation and quality assurance processes are essential components of sustainable POCUS programs, and a robust outline of elements to develop successful programs has been published by the American College of Emergency Physicians (ACEP) [44]. Quality assurance processes, in particular, can be challenging to build and maintain and will often require multidisciplinary support for implementation. Institutions with cardiology and radiology specialists may require their involvement in reviewing POCUS studies to confirm appropriate image acquisition quality and interpretative accuracy. There is no formal definition of quality assurance in POCUS, but there should be an effort made to create a longitudinal educational environment for refinement of POCUS skill. Mechanisms ensuring the oversight of educational objectives should ideally involve multidisciplinary specialist collaboration.

In assessing risk, providers and institutions must also understand local, national, and international laws and regulations related to medical care. There are two main categories of law: civil law and common law. Differences exist regarding assessment and assignment of negligence within these systems [45]. Medical malpractice in a common law system falls under the negligence tort which is summarized as a duty and failure to provide an accepted standard of care resulting in harm [46, 47]. In common law, negligence is assigned to an individual or individuals and results in compensation. A no-fault legal system requires identification of a causal relationship between treatment and injury but does not assign responsibility at the level of the individual [48]. Medical malpractice law in the USA is regulated by individual states, so there are subtle differences in tort negligence laws between states. Similarly, European countries may integrate tort law and no-fault systems in varied ways to optimize system efficiency and may also cap compensation [49–51]. Many countries, including Canada and most of those within Europe, have tort liability assessed by a judge, thereby shortening the time in which a decision is rendered. No-fault systems are practiced outside of the courtroom, and claims in New Zealand are adjudicated by a Medical Board review [52]. Practicing pediatrics, or a subspecialty within pediatrics, also reduces the risk of litigation as a recent survey demonstrated pediatrics as the 24th of 25 specialties in proportion of physicians facing malpractice claims. Yet the mean indemnity payments of malpractice suits were highest in pediatrics [53, 54]. In the USA, 34% of all physicians have been involved in a lawsuit [55]. When we integrate literature regarding malpractice claims with geographic and specialty-specific considerations, pediatric POCUS likely represents a low risk (but not “no riskˮ) practice in any current clinical setting, and risk tolerability is dependent upon local contexts of practice.

## POCUS risk: manage

The approach to *manage* POCUS risk requires developing previously discussed infrastructural elements including technology and equipment, standardized training, image storage solutions, documentation processes, quality assurance methods and pathways to ensure provider competency in POCUS applications (Fig. 1, Table 2). A recent survey of academic pediatric critical care programs in the USA found that over 60% of divisions were performing diagnostic ultrasound in the clinical setting. Yet, despite frequent use of ultrasound, less than 25% of institutions were represented within core infrastructural elements suggested by ACEP [56]. At the time of publication, no division had over 25% of pediatric critical care clinicians credentialed in POCUS applications. Attention to each of these aspects of program development is another step towards protecting patients, providers, and institutions from risk, especially when POCUS will be widely performed by physicians and emerges as a standard of care.

**Table 2:** Point-of-care ultrasound risk and mitigation strategies by programmatic infrastructural element

**Infrastructural element**	**Risk**	**Mitigation strategy**	**Challenges to mitigation**
Technology and equipment	Direct harm to patient from ultrasound and related equipment	Embed as knowledge objectives within educational processes	Limited knowledge of current standards and human data on actual risk
Training	Incompetent in knowledge, psychomotor and/or interpretative educational domains	Development of initial and longitudinal specialty-specific training	Lack of POCUS educational experts No standardized educational curriculum No definition or method of measuring competency
Documentation	Absent or insufficient documentation resulting in a loss of important information from POCUS	Development of POCUS application-specific documentation templates for inclusion in the medical record	Current differences in documentation practice between and within institutions (e.g., paper versus electronic) Identification of appropriate person to interpret and document results
Image storage	Absent or insufficient image storage capabilities resulting in a loss of review capabilities for initial interpretation or longitudinal assessment of changing physiologies	Development of a local POCUS image storage solution	Solutions may not be technologically available in a local clinical environment Storage solutions external to a hospital system (e.g., “cloud-basedˮ) may not be linked to a medical record and may not be viewable to other clinicians
Quality assurance	A lack of a review processes and/or a review process led by unqualified individuals results in the clinical translation of inadequate POCUS skills integrated in patient care	Development of a quality assurance process providing timely feedback to providers across educational domains led by appropriate specialists	Specialists for oversight likely found in other specialties, particularly in the early phases of POCUS program development No definition of “specialistˮ in many POCUS applications Significant time and effort to build multidisciplinary team for the review of images and to create feedback mechanisms
Processes to define and confirm competency (e.g., credentialing)	Absent institutional or national processes for clinical provider integration of POCUS in patient care	Institutional or national POCUS credentialing or certification processes resulting in clinical privileges for providers completing POCUS training	Requires many of the above elements to be in place or actively in development Resistance from administrators with little knowledge of POCUS

The development of programmatic infrastructural elements embedded with risk mitigation strategies likely results in a symbiotic reduction of overall risk given their obvious interdependence with one another

This leads us to the most important element in risk mitigation: *collaboration*. Collaboration requires listening to the concerns expressed by others, whether physicians, administrators, or regulatory agencies. It involves working together to develop shared solutions. Curriculum build and training requires not only ideas and skill development by those within a specialty but also guidance from consultants outside the specialty. The American Society of Echocardiography recently published a statement recognizing and supporting the use of POCUS by adult clinicians and suggested methods by which echocardiography laboratories can assist in program development [57]. Departments and institutions may be unable to support robust quality assurance processes. External support systems may be modeled after telemedicine solutions used by groups such as Médecins Sans Frontières for POCUS quality assurance platforms in remote settings, and information technology growth has resulted in faster and cheaper storage platforms to integrate images and documents in the medical record [58]. Electronic health record solutions will undoubtedly be available to all practice settings if not already present. Finally, development of institutional processes to confirm competency requires collaboration with multi-specialty clinical and administrative leaders and represents institutional investment in safe and high quality pediatric care.

## Conclusion

- Risk analysis of any practice in medicine is a complex calculus incorporating the delivery of care by providers and institutions, the outcomes of patients, and the setting of clinical practice. POCUS use is likely to increase across all pediatric specialties and its translation to clinical care likely represents a low risk, but not no-risk practice. Though a clinician may currently work in an environment with limited medicolegal risk, legal frameworks are likely to evolve with an emerging emphasis on quality and safety across the medical field. We suggest that listening to concerns and partnering with experienced providers, administrators, and regulatory agencies will not only help to develop strategies towards risk reduction, but also result in a practice structure that improves provider performance and patient outcome.

## Data Availability

None.

## Abbreviations

POCUS: Point-of-care ultrasound
ESPR: European Society of Paediatric Radiology
ESEP: European Society of Emergency Pediatrics
ESEM: European Society for Emergency Medicine
BMUS: British Medical Ultrasound Society
ACGME: Accreditation Council for Graduate Medical Education
TNE: Targeted Neonatal Echocardiography
ACEP: American College of Emergency Physicians
AHRQ: Agency for Healthcare Research and Quality
ECRI: Emergency Care Research Institute
